# Chronic Hepatitis B Virus Infection Associated with Increased Colorectal Cancer Risk in Taiwanese Population

**DOI:** 10.3390/v12010097

**Published:** 2020-01-14

**Authors:** Fu-Hsiung Su, Thi Nga Le, Chih-Hsin Muo, Sister Arlene Te, Fung-Chang Sung, Chih-Ching Yeh

**Affiliations:** 1 Department of Family Medicine, Cardinal Tien Hospital, Fu Jen Catholic University, New Taipei City 231, Taiwan; williamsufh1@yahoo.com.tw (F.-H.S.); srarlene@yahoo.com (S.A.T.); 2 School of Medicine, College of Medicine, Fu Jen Catholic University, New Taipei City 242, Taiwan; 3 School of Public Health, College of Public Health, Taipei Medical University, Taipei 110, Taiwan; 4 International Master/PhD Program, College of Medicine, Taipei Medical University, Taipei 110, Taiwan; lengahp0201@gmail.com; 5 Management Office for Health Data, China Medical University Hospital, Taichung 404, Taiwan; b8507006@gmail.com; 6 Department of Health Services Administration, College of Public Health, China Medical University, Taichung 404, Taiwan; 7 Department of Public Health, College of Public Health, China Medical University, Taichung 404, Taiwan; 8 Cancer Center, Wan Fang Hospital, Taipei Medical University, Taipei 116, Taiwan

**Keywords:** colorectal cancer, hepatitis B virus, population-based, case-control study

## Abstract

Chronic hepatitis B virus (HBV) infections and colorectal cancer (CRC) are prevalent in Taiwan. We carried out a population-based case-control study to assess the association between HBV infection and CRC risk. Using the National Health Insurance Research Database of Taiwan, we identified 69,478 newly diagnosed patients with CRC from 2005 to 2011. We further randomly selected 69,478 age- and gender-matched controls without CRC from the same database. Odds ratios (ORs) were calculated to evaluate the association between chronic HBV infection and CRC using a logistic regression analysis. HBV infection was found to be associated with the risk of CRC (OR = 1.27, 95% confidence interval (CI) = 1.20–1.33). This relationship was similar in men and women. Age-specific analysis revealed that the CRC risk associated with HBV decreased with age. The adjusted ORs for patients aged <55, 55–64, and 65–74 years were 1.63 (95% CI = 1.48–1.79), 1.24 (95% CI = 1.13–1.37), and 1.02 (95% = 0.92–1.13), respectively. In conclusion, this study suggests that chronic HBV infection is significantly associated with an increased risk of CRC. Monitoring the risk of CRC development in young patients with HBV infection is crucial.

## 1. Introduction

Colorectal cancer (CRC) is one of the most common cancers and the third leading cause of cancer-related deaths in the world [1]. The incidence rate of CRC has dramatically increased in some Asian countries (such as Japan, China, and Korea) over the past few decades [2]. In Taiwan, the incidence rate of CRC increased by 30% from 2000 to 2016. Moreover, CRC has become the second most common cancer and the second leading cause of cancer-related deaths in the country [3]. Genetic mutation or instability, changes in environmental and lifestyle factors (such as obesity, sedentary lifestyle, smoking, red meat consumption, and aging), and chronic intestinal inflammation are some of the well-established etiologies of CRC [4].

Hepatitis B virus (HBV) is considered to be a hepatotropic virus and is one of the major causes of hepatocellular carcinoma (HCC) worldwide [5]. More than 257 million people worldwide have chronic HBV infections, with the majority of them living in Africa and Asia [6]. Hence, HBV infection contributes to the most serious challenges currently posed by infectious diseases in public health. Taiwan is an endemic area for HBV infection. The chronic HBV carrier rate among the general Taiwanese population ranged from 15% to 20% prior to the introduction of the national HBV vaccination program in 1984 [7]. The nationwide HBV vaccination campaign program has successfully reduced the prevalence rate of chronic HBV to <1.0% in people born after 1992 [8].

Furthermore, studies have indicated the existence of HBV in several extrahepatic organs and tissues, such as the kidneys, colon, stomach, lymph nodes, bone marrow, and pancreas [9,10,11]. Recent studies have suggested that HBV infection is associated with the risk of developing pancreatic cancer [12], gastric cancer [13], intrahepatic cholangiocarcinoma, and non-Hodgkin’s lymphoma [14,15]. The possible association between chronic HBV infection and CRC has been postulated. In a recent retrospective chart review involving 487 patients undergoing screening or diagnostic colonoscopy, the adenoma detection rate was higher in HBV carriers than in the non-HBV population, although this observation did not reach statistical significance. However, the authors observed a positive association between HBV infection and the presence of distal colorectal adenoma [16]. In another study, Kim et al. suggested that HBV infection was independently associated with the development of advanced colorectal adenoma [17]. In a recent Chinese prospective study, Song et al. found that participants with HBsAg seropositive (*N* = 15,355) had a hazard ratio (HR) of 1.42 (95% CI, 1.12–1.81) for colorectal cancer compared with HBsAg seronegative [18].

The association between HBV infections and CRC Taiwanese patients has not been investigated in detail. Moreover, the role of HBV infection as a risk factor for CRC is unclear. Therefore, in the present study, we used the large insurance claims database in Taiwan to conduct a case-control study and assess the CRC risk in patients with HBV infection.

## 2. Materials and Methods

### 2.1. Data Sources

This was a population-based case-control study using data obtained from the NHIRD in Taiwan. The database contained original data from the state-run NHI program, which was established in 1995 and provides national health care for 23 million Taiwanese residents. The coverage rate of this mandatory, single-payer insurance program was up to nearly 99% of the Taiwanese population by the end of 2004 [19]. The details of the program have been presented in our previous papers [20]. This study was approved on April 18, 2012 by the Institutional Review Board of China Medical University and Hospital Research Ethics Committee (IRB approval number: CMU-REC-101-012).

### 2.2. Study Population

Patients newly diagnosed with CRC (International Classification of Diseases, Ninth Revision, Clinical Modification; ICD-9-CM 153–154) from 2005 to 2011 were identified from the RCIP database. The RCIP is one of the patient care programs in Taiwan used to protect insured people with serious diseases and to reduce the financial burden. CRC is an NHI-defined catastrophic illness, and the NHI program covers the costs incurred during the treatment of this disease [21]. The primary care physician of patients with newly diagnosed CRC must submit relevant clinical, laboratory, and imaging information to the NHI administration to qualify for a catastrophic illness certificate.

This study assessed the association between chronic HBV infection (ICD-9-CM 070.2, 070.3, and V02.61) and CRC risk. Patients with human immunodeficiency virus (HIV) were excluded (ICD-9-CM 042, 043, 044, and V08). Additionally, patients with chronic HCV infection (ICD-9-CM 070.41, 070.44, 0.70.51, 0.70.54, and V02.62) were excluded. HBsAg and anti-HCV antibody were the diagnostic serum markers for HBV and HCV infections, respectively. Overall, after excluding 2370 patients (3 patients infected with HIV, 2303 patients infected with HCV alone and coinfected with HBV, and 64 patients with missing information on age and gender), 69,478 CRC patients were enrolled in this study.

**Figure 1:**
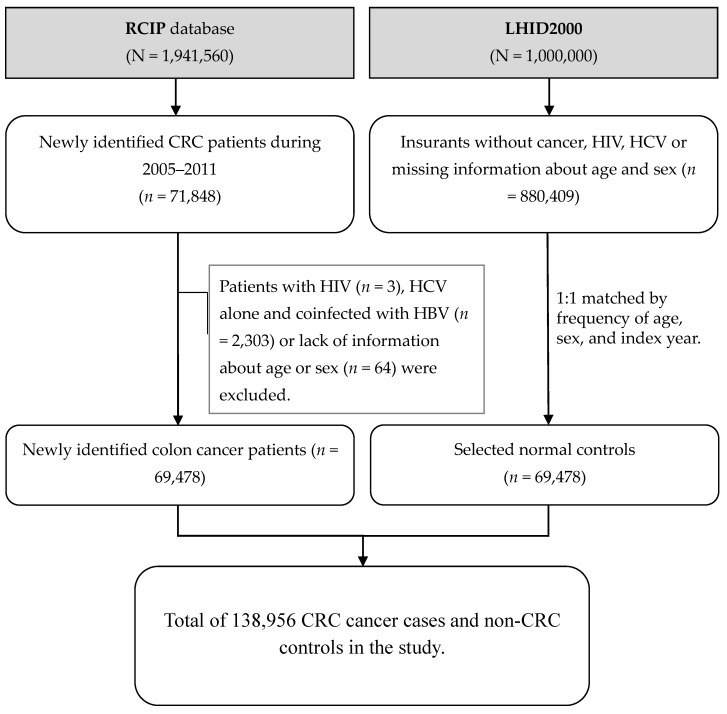
Flow chart for the selection of study patients. Abbreviations: CRC: Colorectal cancer; HCV: Chronic hepatitis C infection; HIV: Human immunodeficiency virus; LHID2000: Longitudinal Health Insurance Database 2000; RCIP: Registry for Catastrophic Illness Patient.

Controls were randomly selected from the Longitudinal Health Insurance Database 2000 (LHID2000), which contains all claims data of one million randomly selected people in the NHIRD and updated registries from 2000 to 2011. The control group comprised randomly selected people, without a history of CRC, HCV, and/or HIV, who were matched to the CRC patients by age and gender at a ratio of 1:1. In this study, the age of each individual was calculated as the interval between the index date and the date of birth. Among the 880,409 people eligible as controls, 69,478 were enrolled in this study. The flowchart for the recruitment of the CRC patients from the RCIP database and the controls from the LHID2000 is illustrated in Figure 1.

### 2.3. Statistical Analyses

The chi-square test was used to compare the distributions of the demographic characteristics (age, occupation, monthly income, and geographical location, as well as the urbanization level of the area of residence) and comorbidities between the CRC patients and controls. We selected 528 USD (USD: 1 USD as 30 new Taiwanese dollars) and 833 USD as the cutoff points for the monthly income. Associated comorbidities, such as diabetes mellitus, hypertension, hyperlipidemia, CAD, renal disease, COPD, obesity, and liver cirrhosis diagnosed during 2005–2011 were ascertained using diagnostic ICD-9-CM codes.

A multivariable logistic regression analysis was applied to calculate the aORs and 95% CIs for variables significantly associated with CRC risk. Furthermore, the analyses of the association between chronic HBV infection and CRC risk were stratified by age and gender, and the odds and ORs of HBV infection were estimated. A two-sided *p* value <0.05 was considered statistically significant. Statistics were analyzed using SAS statistical software (version 9.4 for Windows; SAS Institute, Inc., Cary, NC, USA).

## 3. Results

### 3.1. General Characteristics of Patients

Table 1 lists the demographic characteristics of the CRC patients (*N* = 69,478) and controls (*N* = 69,478). The disease was more common in men (57.2% in both groups) and in individuals aged >50 years (86.9%). The distributions of geographical region, occupation, urbanization level, and monthly income were significantly different between the CRC patients and the controls (*p* < 0.05). Diabetes mellitus and hypertension were more prevalent in the CRC patients than in the controls (*p* < 0.001), whereas coronary artery disease (CAD), chronic obstructive pulmonary disease (COPD), and liver cirrhosis were more prevalent in the controls than in the CRC patients (*p* < 0.05).

### 3.2. Overall Risk of CRC in Patients with Chronic HBV Infection

Table 2 shows that CRC patients were more likely to be HBV-positive than the controls (5.09% vs. 4.21%) with a crude odds ratios (ORs) of 1.22 (95% confidence interval (CI) = 1.16–1.28). The adjusted OR (aOR) was 1.27 (95% CI = 1.20–1.33) after controlling for demographic characteristics and comorbidities in the multivariable logistic regression analysis. HBV carriers without HDV were significantly associated with CRC (aOR = 1.27 (95% CI = 1.21–1.34)). No significant relationship was found for those with coinfection.

### 3.3. Age-Specific Risk of CRC in Patients with Chronic HBV Infection

The age-specific analysis revealed that the odds of HBV infection declined with an increase in age in both the CRC patients and controls (Table 3). The differences in the odds between the two groups were greater at <55 years and between 55 and 64 years (0.034 and 0.012, respectively), but similar at 65–74 and >75 years. Compared with the controls, the incidence of HBV in the CRC patients belonging to these two age groups (<55 years and between 55 and 64 years) was higher with aORs of 1.63 (95% CI = 1.48–1.79) and 1.24 (95% CI = 1.13–1.37), respectively. The effect of the interaction between the HBV status and age on the risk of CRC was statistically significant (*p* < 0.001).

### 3.4. Sex-Specific Risk of CRC in Patients with Chronic HBV Infection 

Table 4 shows that the sex-specific odds of chronic HBV infection were greater in the CRC patients and in men than in the controls and women, respectively. However, the aORs of CRC as HBV carriers were similar for women (1.29, 95% CI = 1.18–1.40) and men (1.25, 95% CI = 1.17–1.34). The effect of the interaction between the HBV status and gender on CRC risk was not statistically significant (*p* = 0.569).

We also performed a sensitivity analysis to evaluate how the chronic HBV infection was associated with risks of colon and rectal cancers. Colon cancer patients had the highest infection rates with an aOR of 1.32 (95% CI = 1.24–1.41), followed by rectal cancer with an aOR of 1.17 (95% CI = 1.08–1.28; Table S1). This relationship was stronger in young CRC patients aged <55 years with aORs of 1.76 (95% CI = 1.56–2.00) for colon cancer and 1.44 (95% CI = 1.23–1.67) for rectal cancer (Table S2).

## 4. Discussion

In this large-scale, population-based case-control study in an endemic area of chronic HBV infection, we found that the risk of CRC was significantly associated with chronic HBV infection, particularly in younger CRC patients. Approximately 19% of the global cancer burden can be linked to five infectious agents, namely Epstein–Barr virus, human papillomaviruses, HBV, HCV, and *Helicobacter pylori* [22]. HBV, an enveloped DNA virus from the hepadnavirus family, has a high affinity for hepatocytes. In most HBV-endemic regions like Taiwan, the infection occurs mainly during early childhood and through mother-to-infant transmission, which accounts for approximately 50% of the chronic infection cases [23]. 

HBV can be integrated into the human genome, leading to genomic instability and hepatocarcinogenesis. Following HBV entry into hepatocytes, the relaxed circular DNA (rcDNA) or, more rarely, the double-stranded linear DNA (dslDNA) genome of HBV is released into the cytoplasm and transported to the nucleus. The intra-nuclear dslDNA HBV genomes can then be integrated into the host cell genome at the site of double-stranded DNA breaks via DNA repair pathways [24]. Integrated viral sequences are essential for the production of mutated HBx or preS2/S proteins, contributing to tumorigenesis [25]. The promotion of genomic instability as the result of both the integration of viral DNA into the host genome and the activity of viral proteins is one of the mechanisms that have been reported [25,26,27,28]. Another direct mechanism of HBV carcinogenesis is based on the ability of viral proteins (HBx, HBc, and preS) to affect cell functions, including cell proliferation and cell viability, and to sensitize liver cells to mutagens [25,26,27,28]. The third reported mechanism is the insertional mutagenesis, which can integrate the viral DNA into host cancer genes (telomerase reverse transcriptase (TERT), myeloid/lymphoid or mixed-lineage leukemia 4 (MLL4), and cyclin E1 (CCNE1)). HBV can thus promote heptocarcinogenesis [25,26,27,28]. Previous studies have indicated that patients with HBV integration in the TERT gene had significantly poorer survival rates [28]. In HBV-related HCCs, telomerase is reactivated in more than 90% of cases due to HBV insertion in the TERT promoter (10–15%) or somatic TERT promoter mutations (54–60%). TERT re-expression can also cause a direct transcriptional activation of the TERT promoter by the wild-type HBx protein as well as truncated HBx and MHBst proteins [27].

As HBV DNA integrates in the host genome, HBx colocalizes in the mitochondrial cytoplasm and nucleus. In the cytoplasm, HBx regulates protein degradation, cellular transcription, apoptosis, and cell proliferation. HBx stimulates the replication of HBV in the nucleus [25]. There are five suggested mechanisms through which HBx induces parthenogenesis [29]. First, HBx interferes with nucleotide excision repair, which leads to DNA damage through both p53-dependent and -independent pathways [30,31]. Second, HBx protein also acts on anti- and pro-apoptotic pathways, particularly important in the inhibition of p53 [30,31,32]. Third, HBx protein may increase the expression of TERT and telomerase activity by which it enhances the life-span of hepatocytes and contributes to hepatocarcinogenesis [31]. Furthermore, HBx protein modulates the transcription of methyltransferases, which can lead to both regional hypermethylation of DNA, resulting in the silencing of tumor suppressor genes, and global hypomethylation, resulting in chromosomal instability [31]. Finally, HBx protein acts as a potent transactivator and regulates transcriptional activity via direct protein–protein interaction. The transactivation functions of HBx are carried out in cytoplasm by signaling pathways and in the nucleus by DNA-binding proteins [29]. Cancer development typically occurs after 20–30 years of infection [22].

Mutagenic activity is an important factor in the development of malignancy. HBV has been identified in the colonic mucosa [9]. However, studies on the association between HBV infection and CRC risk are limited [16,17,18,33]. Fahal et al. reported that the detection rate of hepatitis B surface antigen (HBsAg) among patients with colonic carcinoma was 8% [33]. Kim et al. revealed that HBV infection and HBV DNA are independently associated with advanced colorectal adenoma development [17]. A retrospective chart review was performed on 588 consecutive patients undergoing screening or diagnostic colonoscopy, and a positive association between HBV infection and the presence of distal colorectal adenomas was reported (OR = 2.16, 95% CI = 1.06–4.43, *p* = 0.04) [16]. A recent Chinese prospective study in 15,355 HBsAg seropositive participants also found a 42% higher risk of colorectal cancer [18].

Chronic inflammation, such as ulcerative colitis and Crohn’s disease, is one of the major risk factors for the development of CRC [4]. Chronic HBV infection can lead to HCC through inflammation related to chronic active hepatitis [34]. Additionally, HBV can exist in the colonic mucosa [9]; therefore, CRC can be highly postulated to be more likely to develop in patients with chronic HBV infection. In addition, the development of cancer through infection cascades may occur two to three decades post-HBV infection [22]. Taiwan might be considered a potentially ideal region to observe this phenomenon because many of the patients with chronic HBV are infected during neonatal development or early childhood [23]. Hence, HBV may play a role in the development of CRC through a process that is similar to that seen during the development of HBV-related HCC. Chronic inflammation related to active hepatitis causes oxidative DNA damage and leads to p53 mutation. HBx can bind to p53 and interfere with its role in DNA damage repair, and consequently, in the transformation of colorectal adenoma into carcinoma [34]. In addition, chronic HBV infection was also reported to affect the modulation of the host immune system [35], which may also play a role in the development of CRC.

One study revealed that African Americans have a 20% higher incidence of colon cancer than Caucasians [36]. Forde et al. have reported a fourfold higher HBV infection rate in African Americans than in Caucasians [37]. Agrawal et al. also found that African Americans were diagnosed with CRC at a younger age compared with Caucasians [38]. The findings of the present study also showed that CRC patients who were diagnosed at a younger age were more likely to be HBV-positive, which mirrors the current trend in some other Asian countries. The incidence of overall CRC, particularly among those with early-onset disease, has dramatically increased in the past few decades, particularly in Korea where more than a twofold increase has been reported [2,39]. These studies have suggested that in spite of well-established genetic, environmental, and lifestyle risk factors, chronic infection associated with HBV may play an important role in the development of CRC.

This study has several limitations. One is that some HBV-infected patients without obvious clinical symptoms might not receive medical services. As a result, some of these patients might be classified as non-HBV-infected. However, CRC diagnosis is unrelated to the HBV status. This nondifferential misclassification may lead to a bias of the estimated OR toward the null value. Second, patients with CRC, chronic HBV hepatitis, and comorbidities were identified using ICD-9-CM diagnosis codes, which are less accurate than clinical diagnoses. However, the National Health Insurance (NHI) administration randomly samples a fixed percentage of claims from every hospital. In addition, patients are randomly interviewed and charts are reviewed each year to verify the validity of the diagnoses and the quality of care. Patients with confirmed CRC diagnosis in Taiwan receive a catastrophic illness certificate, and the NHI program covers the treatment costs incurred by the disease. Therefore, these patients are representative of the CRC population in Taiwan. Third, information on some important factors that might be related to CRC (such as red meat consumption, environmental exposure, obesity, cigarette smoking, sedentary lifestyle, and family history of CRC) were not available in the NHI Research Database (NHIRD) and Registry for Catastrophic Illness Patient (RCIP) of Taiwan. To compensate for the lack of lifestyle-related data, the study accounted for related chronic diseases. COPD is correlated with the dose levels of cigarette smoking. Likewise, CAD, hypertension, and hyperlipidemia can be the results of poor diet and lifestyle. However, even with these adjustments, some of the potential confounding effects associated with these factors cannot be ruled out. Fourth, data of HBV DNA counts are not currently available in the NHIRD and RCIP of Taiwan; hence, comparisons of HBV viremia between case and control groups are not made. Finally, the vast majority of the residents in Taiwan are of Chinese ethnicity. Therefore, the ability to generalize the results to other racial/ethnic groups is unclear, given that the transmission route of HBV infection in the Chinese population might not be the same as that in other ethnic groups.

## 5. Conclusions

In summary, this population-based case-control study suggests that chronic HBV infection is associated with the development of CRC, particularly at a young age. Further studies are required to apply our findings to other regions or races and to clarify the underlying pathophysiological mechanisms involved in the association between chronic HBV infection and CRC.

## Figures and Tables

**Table 1:** Comparison of demographic characteristics and comorbidities between colorectal cancer patients and controls.

Variables	Controls (N = 69,478)		Cases (N = 69,478)		
	*n*	(%)	*n*	(%)	*p* Value *
Sex					1.000
Women	29,750	(42.8)	29,750	(42.8)	
Men	39,728	(57.2)	39,728	(57.2)	
Age, years					1.000
<20	37	(0.05)	37	(0.05)	
20–29	484	(0.70)	484	(0.70)	
30–39	2252	(3.24)	2252	(3.24)	
40–49	6347	(9.14)	6347	(9.14)	
50–59	14,545	(20.9)	14,545	(20.9)	
60–69	16,440	(23.7)	16,440	(23.7)	
70–79	18,304	(26.4)	18,304	(26.4)	
≥80	11,069	(15.9)	11,069	(15.9)	
Geographical region					<0.001
Northern	29,565	(42.6)	29,740	(42.8)	
Central	14,241	(20.5)	13,803	(19.9)	
Southern	21,763	(31.3)	22,535	(32.4)	
Eastern and islands	3909	(5.63)	3400	(4.89)	
Occupation					<0.001
White collar	32,227	(46.4)	33,665	(48.5)	
Blue collar	29,023	(41.8)	28,421	(40.9)	
Retired and others	8228	(11.8)	7392	(10.6)	
Urbanization level					<0.001
Urban	18,776	(27.0)	19,396	(27.9)	
Suburban	31,055	(44.7)	31,778	(45.7)	
Rural	19,647	(28.3)	18,304	(26.4)	
Monthly income, USD					0.011
<528	21,815	(31.4)	21,894	(31.5)	
528–832	33,119	(47.7)	32,640	(47.0)	
≥833	14,544	(20.9)	14,944	(21.5)	
Comorbidities					
Diabetes	16,706	(24.1)	19,165	(27.6)	<0.001
Hypertension	36,709	(52.8)	38,189	(55.0)	<0.001
Hyperlipidemia	22,072	(31.8)	22,029	(31.7)	0.804
CAD	19,995	(28.8)	19,464	(28.0)	0.002
Renal disease	10,898	(15.7)	10,998	(15.8)	0.462
COPD	26,943	(38.8)	24,707	(35.6)	<0.001
Obesity	1055	(1.52)	1055	(1.52)	1.000
Liver cirrhosis	16,166	(23.3)	15,845	(22.8)	0.041

* Chi-square test. Abbreviations: CAD: Coronary artery disease; COPD: Chronic obstructive pulmonary disease; N: Number; 1 USD to 30 NTD. Some potential risk factors for CRC (red meat consumption, environmental exposure, cigarette smoking, sedentary lifestyle, and family history of CRC) were not available in the insurance claims data. COPD, CAD, hypertension, and hyperlipidemia, which are the results of these potential risk factors, were used to perform adjustment in this study.

**Table 2:** Comparison of chronic hepatitis B virus infection between colorectal cancer cases and controls.

HBV	Controls		Cases		Crude		Adjusted	
	*n*	(%)	*n*	(%)	OR (95% CI)	*p* Value	OR (95% CI) ^b^	*p* Value
No	66,554	(95.8)	65,942	(94.9)	1.00 (ref)		1.00 (ref)	
Yes ^a^	2924	(4.21)	3536	(5.09)	1.22 (1.16–1.28)	<0.001	1.27 (1.20–1.33)	<0.001
HBV only	2722	(3.92)	3315	(4.77)	1.23 (1.17–1.29)	<0.001	1.27 (1.21–1.34)	<0.001
HBV + HDV	202	(0.29)	221	(0.32)	1.10 (0.91–1.34)	0.31	1.15 (0.95–1.39)	0.16

^a^ Patients infected with HIV, HCV alone, and dual HBV + HCV were excluded. ^b^ Adjusted for geographical region, occupation, urbanization level, monthly income, diabetes, hypertension, CAD, COPD, liver cirrhosis, age, and sex (significant variables in Table 1). Abbreviations: HBV: Chronic hepatitis B virus infection; HDV: Hepatitis D virus infection; OR: Odds ratio; ref: Reference.

**Table 3:** Age-specific odds of chronic hepatitis B virus infection and case-to-control adjusted odds ratio of the infection.

Age	HBV	Controls	Cases	Adjusted OR (95% CI) ^a^	*p* Value
<55	No	14,900	14,439	1.00 (ref)	
	Yes	808	1269	1.63 (1.48–1.79)	<0.001
	Odds	0.054	0.088		
55–64	No	14,773	14,618	1.00 (ref)	
	Yes	847	1002	1.24 (1.13–1.37)	<0.001
	Odds	0.057	0.069		
65–74	No	17,184	17,188	1.00 (ref)	
	Yes	762	758	1.02 (0.92–1.13)	0.739
	Odds	0.044	0.044		
≥75	No	19,697	19,697	1.00 (ref)	
	Yes	507	507	1.03 (0.91–1.17)	0.611
	Odds	0.026	0.026		

^a^ Adjusted for geographical region, occupation, urbanization level, monthly income, diabetes, hypertension, CAD, COPD, liver cirrhosis, and sex (significant variables in Table 1). Abbreviations: HBV: Chronic hepatitis B infection; OR: Odds ratio; ref: Reference.

**Table 4:** Sex-specific odds of chronic hepatitis B virus infection and case-to-control adjusted odds ratio of the infection.

Sex	HBV	Controls	Cases	Adjusted OR (95% CI) ^a^	*p* Value
Women	No	28,668	28,416	1.00 (ref)	
	Yes	1082	1334	1.29 (1.18–1.40)	<0.001
	Odds	0.038	0.047		
Men	No	37,886	37,526	1.00 (ref)	
	Yes	1842	2202	1.25 (1.17–1.34)	<0.001
	Odds	0.049	0.059		

^a^ Adjusted for geographical region, occupation, urbanization level, monthly income, diabetes, hypertension, CAD, COPD, liver cirrhosis, and sex (significant variables in Table 1). Abbreviations: HBV: Chronic hepatitis B infection; OR: Odds ratio; ref: Reference.

## Supplementary Materials

Supplementary materials can be found at https://www.mdpi.com/1999-4915/12/1/97/s1. All data are available from the NHIRD of Taiwan (http://nhird.nhri.org.tw/). Requests for data can be sent as a formal proposal to the NHIRD. Table S1. Risk of colorectal cancer associated with chronic hepatitis B virus infection stratified by tumor location. Table S2. Age-specific risk of colorectal cancer associated with chronic hepatitis B virus infection stratified by tumor location.
