# Supplementary material for: Chronic Hepatitis B Virus Infection Associated with Increased Colorectal Cancer Risk in Taiwanese Population
Source: Viruses. 2020 Jan 14;12(1):97. doi: 10.3390/v12010097 (PMC7019239; doi:10.3390/v12010097)
Supplement: Supplementary file 1 [file viruses-12-00097-s001.pdf]

Table S1. Risk of colorectal cancer associated with chronic hepatitis B virus infection stratified by tumor location.

	Controls		Cases		Crude		Adjusted	
HBV	n	(%)	n	(%)	OR (95% CI)	*p* value	OR (95% CI) [a]	*p* value
Colon								
No	40583	(95.8)	40074	(94.6)	1.00 (ref)		1.00 (ref)	
Yes	1784	(4.21)	2293	(5.41)	1.30 (1.22-1.39)	<0.001	1.32 (1.24-1.41)	<0.001
Rectum								
No	25971	(95.8)	25868	(95.4)	1.00 (ref)		1.00 (ref)	
Yes	1140	(4.20)	1243	(4.58)	1.10 (1.01-1.19)	0.031	1.17 (1.08-1.28)	<0.001

[a] Adjusted for geographical region, occupation, urbanization level, monthly income, diabetes, hypertension, CAD, COPD, liver cirrhosis, age, and sex

Table S2. Age-specific risk of colorectal cancer associated with chronic hepatitis B virus infection stratified by tumor location.

HBV	Age<55			Age 55–64			Age 65–74			Age ≥75		
	Co/Ca	OR (95%CI)[a]	*p* value	Co/Ca	OR (95%CI)[a]	*p* value	Co/Ca	OR (95%CI)[a]	*p* value	Co/Ca	OR (95%CI)[a]	*p* value
Colon												
No	8767/8433	1.00 (ref)		8713/8601	1.00 (ref)		10411/10366	1.00 (ref)		12692/12674	1.00 (ref)	
Yes	465/799	1.76 (1.56-2.00)	<0.001	542/654	1.23 (1.09-1.39)	0.001	446/491	1.09 (0.96-1.25)	0.191	331/349	1.08 (0.93-1.26)	0.317
Rectum												
No	6133/6006	1.00 (ref)		6060/6017	1.00 (ref)		6773/6822	1.00 (ref)		7005/7023	1.00 (ref)	
Yes	343/470	1.44 (1.23-1.67)	<0.001	305/348	1.27 (1.08-1.50)	0.004	316/267	0.90 (0.75-1.06)	0.200	176/158	0.93 (0.75-1.17)	0.548

[a] Adjusted for geographical region, occupation, urbanization level, monthly income, diabetes, hypertension, CAD, COPD, liver cirrhosis, age, and sex
OR: odds ratio; CI, confidence interval; Co/Ca: Controls/Cases.
